# Unexpected Position of a Hemodialysis Catheter

**DOI:** 10.7759/cureus.117676

**Published:** 2026-10-09

**Authors:** João Figueiredo Martins, Ana Sofia Domingos, Luís Gaião Santos, Paula Guimarães

**Affiliations:** 1 Nephrology, Unidade Local de Saúde São José, Lisbon, PRT

**Keywords:** catheter malposition, central venous catheterization, congenital venous anomalies, hemodialysis catheter, partial anomalous pulmonary venous return, vascular access

## Abstract

Central venous catheterization is frequently employed to obtain urgent hemodialysis access, and catheter malposition due to unrecognized venous anomalies is an underappreciated complication. We describe the case of a 77-year-old man with end-stage renal disease and an extensive vascular access history who required a temporary hemodialysis catheter after thrombosis of his arteriovenous graft. The catheter was placed uneventfully in the left internal jugular vein under ultrasound guidance, but control radiography showed its tip lying in the left hemithorax, and blood gas analysis from the catheter was compatible with arterial blood. Contrast-enhanced computed tomography revealed the catheter tip within the left superior pulmonary vein, which drained into the left brachiocephalic vein, consistent with partial anomalous pulmonary venous return, a rare congenital anomaly usually discovered incidentally. The catheter was removed without complications, and the patient was managed without hemodialysis until his arteriovenous graft was successfully salvaged two days later. This case highlights that an atypical catheter trajectory combined with an arterialized blood gas does not imply arterial puncture and that anomalous venous anatomy should be in the differential diagnosis.

## Introduction

Central venous catheterization remains the most common method of obtaining urgent vascular access for hemodialysis, with the internal jugular vein often preferred for its lower complication rate compared with subclavian or femoral approaches. Although generally safe when performed under ultrasound guidance, the procedure can be associated with complications, and catheter malposition due to unrecognized venous anomalies is an underappreciated pitfall [1].

Partial anomalous pulmonary venous return (PAPVR) is a rare congenital defect in which one or more pulmonary veins drain into a systemic vein or directly into the right atrium instead of the left atrium [2]. It may affect either side and is only rarely bilateral. Most cases are right-sided, typically draining into the superior vena cava or the right atrium, whereas left-sided forms usually drain into the left brachiocephalic vein through an anomalous vertical vein [3]. This congenital anomaly is usually asymptomatic and often found only incidentally, on imaging obtained for other reasons [4].

We report a case of a hemodialysis catheter that followed an unusual course on control radiography and yielded arterialized blood on blood gas sampling, leading to the incidental diagnosis of PAPVR connecting with the left brachiocephalic vein. This case illustrates the diagnostic reasoning required when catheter positioning and blood gas findings are discordant with expected venous placement, and underscores the implications of congenital central venous anomalies for hemodialysis access planning.

## Case presentation

A 77-year-old man with end-stage renal disease and a long-standing vascular access history was referred for hemodialysis catheter placement following arteriovenous graft thrombosis. Relevant medical history included chronic kidney disease on hemodialysis for approximately nine years, type 2 diabetes mellitus, arterial hypertension, coronary artery disease, and prostate cancer under surveillance. His vascular access history was extensive, including tunneled catheters in both internal jugular veins and bilateral brachiocephalic fistulas, followed by a left brachial-axillary graft created approximately four years earlier, which was his current access. On admission, ultrasound revealed a thrombosed right internal jugular vein but a patent left internal jugular vein, and a 20 cm temporary hemodialysis catheter was placed uneventfully under ultrasound guidance.

Control chest radiography (Figure 1), however, showed catheter malposition, with the tip located in the left hemithorax. This raised concern for possible arterial catheterization, venous perforation into the mediastinum or pleural space, or catheter progression into a tributary vein (such as the left internal thoracic vein or the accessory hemiazygos vein). Blood drawn from the catheter for gas sampling showed a partial pressure of oxygen of 70 mmHg, compatible with arterial blood.

**Figure 1:**
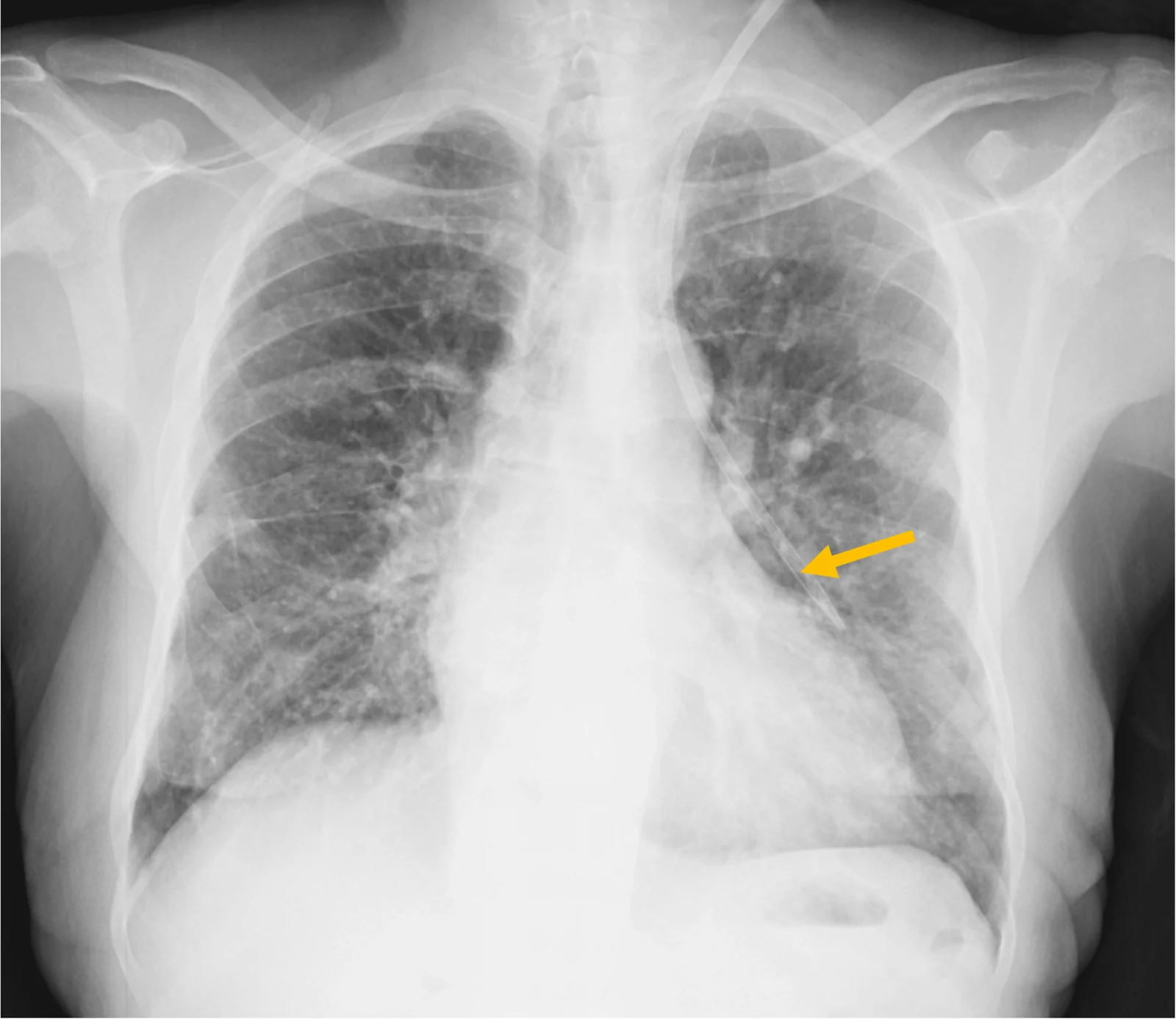
Chest radiography showing malpositioned left internal jugular vein catheter The arrow indicates the catheter tip in the left hemithorax.

Urgent contrast-enhanced computed tomography (CECT) showed the catheter tip within the left superior pulmonary vein (Figure 2), which drained directly into the left brachiocephalic vein rather than the left atrium, establishing the diagnosis of PAPVR.

**Figure 2:**
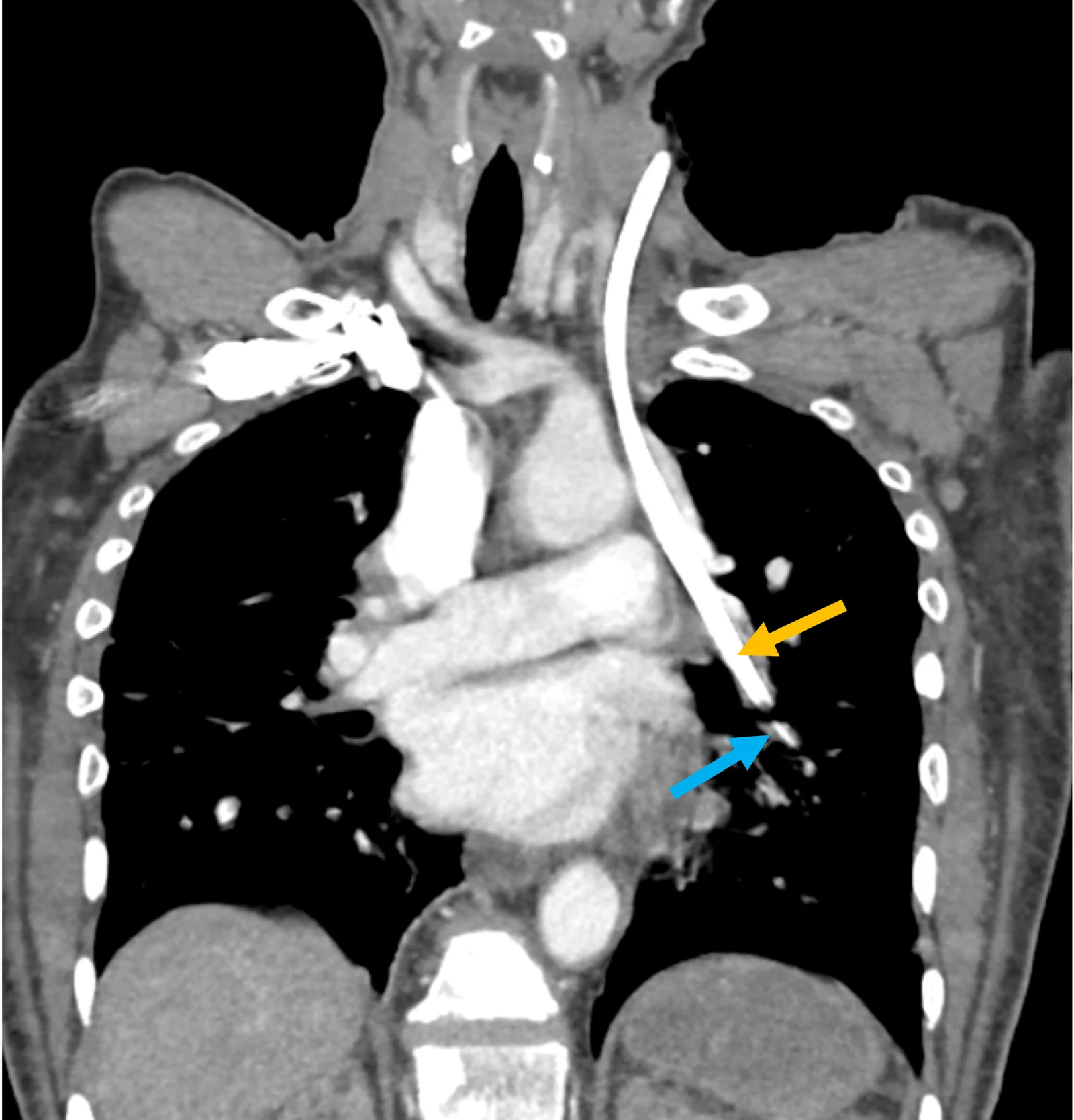
Contrast-enhanced computed tomography showing the catheter within the left superior pulmonary vein, which drains into the left brachiocephalic vein The yellow arrow indicates the catheter, and the blue arrow indicates the left superior pulmonary vein.

Following consultation with thoracic surgery, the catheter was removed without complications, and the patient remained asymptomatic throughout. The remainder of the hospital stay was unremarkable. The patient was managed without hemodialysis until his graft was successfully salvaged two days later by percutaneous hydrodynamic thrombectomy with heparinized saline. A stenosis at the graft-vein anastomosis was identified and treated with balloon angioplasty. Graft patency was restored, and the patient resumed hemodialysis through it. At three months of follow-up, the graft remained patent.

## Discussion

PAPVR is a rare central venous anomaly, with a reported prevalence of 0.4%-0.7% in adults [5]. Among left-sided forms, drainage of the left superior pulmonary vein into the left brachiocephalic vein is the most frequent variant [3]. Because the left-to-right shunt is usually small, most cases remain silent for life and are found only incidentally on cross-sectional imaging obtained for unrelated reasons [4].

Mechanical complications of central venous catheter placement include arterial puncture, hematoma, pneumothorax, arrhythmias, and catheter malposition [1]. Congenital central venous anomalies, such as PAPVR, are an overlooked cause of catheter malposition [6-9].

Accordingly, an unexplained deviation of catheter trajectory on control chest radiography is not always due to arterial catheterization or venous wall perforation, and should raise suspicion for the possibility of an anomalous vein. Furthermore, aspiration of arterial blood from the catheter does not, by itself, confirm arterial cannulation [10], and should be interpreted alongside imaging rather than triggering immediate catheter withdrawal or invasive correction in a hemodynamically stable patient. CECT was decisive in establishing the diagnosis by identifying the anomalous connection between a pulmonary vein and a central vein, as well as excluding alternative explanations for malposition such as arterial puncture or extravascular placement.

A recent report described a similar finding in a patient with acute kidney injury undergoing first-time catheter placement [8]. Our case differs in that it occurred in a patient with established end-stage renal disease and an extensive vascular access history, in whom preservation of remaining access sites was a clinical concern, and whose anomaly had gone undetected through multiple prior ipsilateral catheterizations. This suggests that the anomaly can escape detection even after repeated instrumentation of the same vessel, and that clinicians should not lower their suspicion in patients with a long, apparently uneventful catheterization history. Once the diagnosis was established, catheter removal was uncomplicated, and the patient remained asymptomatic, consistent with the largely benign natural history of this anomaly when unassociated with other structural cardiac disease.

## Conclusions

Congenital venous anomalies, though rare, should be part of the differential diagnosis whenever central venous access behaves atypically, regardless of previous successful catheterizations. An unexpected catheter trajectory with an arterialized blood gas warrants cross-sectional imaging before arterial injury is assumed. In patients with a known or suspected anomaly, or after an episode such as this one, fluoroscopic guidance is recommended for subsequent catheter placement to allow real-time visualization of the catheter tip trajectory and reduce the risk of recurrent malposition.
